# Longitudinal investigation of psychological adaptation and its determinants in primary caregivers following thyroid cancer surgery

**DOI:** 10.3389/fmed.2026.1810443

**Published:** 2026-05-29

**Authors:** Juhua Li, Qian Zhou

**Affiliations:** Department of Thyroid Surgery, West China Hospital, Sichuan University/West China School of Nursing, Sichuan University, Chengdu, Sichuan, China

**Keywords:** caregiver burden, caregiving competence, primary caregivers, psychological adaptation, thyroid cancer

## Abstract

**Objective:**

This study aimed to explore the psychological adaptation status of primary caregivers of postoperative thyroid cancer patients and its influencing factors, and to analyze the relationships among caregiver burden, caregiving competence, and psychological adaptation.

**Methods:**

A 6-month longitudinal survey was conducted with 398 patient-caregiver dyads across four time points: T0 (1 day before discharge), T1 (1 month postoperatively), T2 (3 months postoperatively), and T3 (6 months postoperatively). Psychological adaptation was measured using the Psychological Adaptation Scale (PAS), caregiver burden with the Zarit Burden Interview (ZBI), and caregiving competence with the Family Caregiver Task Inventory (FCTI). Statistical analyses included repeated-measures ANOVA, Pearson correlation, and Generalized Estimating Equations (GEE).

**Results:**

A total of 398 questionnaires were distributed, of which 387 were valid, yielding a response rate of 97.23%. The psychological adaptation scores of caregivers increased significantly from T0 to T3 (*P* < 0.001), while scores on the FCTI and ZBI decreased significantly. Correlation analyses showed that PAS was negatively correlated with both FCTI and ZBI at all-time points (T0–T3). GEE analysis revealed that time (β = 7.928) and having one caregiver (β = 1.035) were significant positive predictors of psychological adaptation (*P* < 0.05). The ZBI score (β = -0.147) and the FCTI score (β = -0.395) were significant negative predictors (*P* < 0.001). Hierarchical regression analysis revealed that in Model 1, monthly per capita income > 5,000 yuan (β = 0.412) and having a carer (β = 0.328, 0.085) were positive predictors of PAS, explaining 18.6% of the variance; After including ZBI, the model explained 36.4% of the variance, with ZBI negatively predicting PAS (β = -0.286); following the further inclusion of FCTI, the final model explained 51.2% of the variance, with both FCTI (β = -0.368) and ZBI (β = -0.271) serving as significant negative predictors (both *P* < 0.001).

**Conclusion:**

The psychological adjustment levels of primary carers of patients who have undergone thyroid cancer surgery showed a significant upward trend in the 6 months following the operation, whilst their caregiving capacity and caregiving burden showed a significant downward trend over the same period. Caregivers who have less capacity to care and who are under more of a burden tend to have a poorer psychological adjustment.

## Introduction

Thyroid cancer incidence has been steadily increasing globally and has become one of the most common malignant tumors, with an annual growth rate of approximately 5% (1). It is estimated that a large number of new cases are diagnosed annually in China, which means that many family caregivers are directly involved in the postoperative rehabilitation of patients (2). With advancements in diagnostic and therapeutic technologies, the survival rate of thyroid cancer patients has significantly increased, but surgeries, subsequent radioactive iodine treatments, thyroid hormone suppression therapy, and other interventions still impose a range of physical and psychological challenges on patients, such as neck scars, voice changes, calcium metabolism disorders, long-term medication-related psychological burden, and fear of recurrence (3). This rehabilitation process places sustained and higher demands on the family support system, with primary caregivers playing a critical role. The mental and physical health of the primary caregivers directly impacts the patient’s recovery and quality of life (4). In contrast to caregivers of high-disability diseases such as stroke, the caregiving scenario for thyroid cancer primary caregivers is unique. On one hand, due to the relatively favorable prognosis of the disease, the pressure they face is less intense (5). On the other hand, the social perception of “small cancer” may lead to their burden being underestimated by friends, family, and even healthcare professionals, resulting in inadequate emotional support and recognition (5, 6). At the same time, postoperative functional symptoms, such as hoarseness, hand and foot numbness, and neck tightness, along with the need for lifelong thyroid hormone replacement therapy, constitute long-term and detailed caregiving demands that test the caregiver’s patience, knowledge, and adaptability (7).

Psychological adaptation refers to the process by which individuals regain psychological balance and achieve functional recovery through cognitive, emotional, and behavioral adjustments when facing significant life events (8). Psychological adaptation has been widely recognized as one of the core health outcomes in cancer caregiving. Studies on caregivers of cancer, including breast cancer have shown that psychological adaptation is significantly negatively correlated with caregivers’ depression and anxiety, and it positively predicts their quality of life and continued caregiving willingness (9, 10). Caregiver burden (Zarit Burden Interview, ZBI) reflects the intensity of physical, emotional, social, and financial stress experienced by caregivers due to their caregiving role (11) Numerous studies have shown that higher caregiver burden is one of the most stable risk factors for predicting anxiety, depression, and deterioration in physical and mental health in caregivers of dementia, stroke, and various cancers (12–14). Caregiving competence (Fulfillment of Caregiving Tasks Inventory, FCTI) reflects caregivers’ positive evaluation of their ability to manage the knowledge, skills, and resources necessary to complete caregiving tasks, acting as a protective psychological resource (15). Research indicates that ZBI and FCTI can influence psychological adaptation levels (16). For instance, in high-burden situations, caregivers with high competence may exhibit greater psychological resilience (17).

However, the interaction between caregiver burden and caregiving competence over time, and how they influence psychological adaptation after thyroid cancer surgery, remains underexplored. Furthermore, the factors that influence the psychological state of primary caregivers are still unclear. Therefore, this study aims to survey primary caregivers of thyroid cancer postoperative patients to understand their psychological health status and analyze related influencing factors, in order to develop targeted interventions that could improve the quality of life for patients and their families.

## Materials and methods

### Study population

This study enrolled thyroid cancer patients and their primary caregivers receiving treatment at West China Hospital of Sichuan University between March 2021 and June 2024. Participants were consecutively recruited during their hospitalization for surgery, and researchers provided them with detailed explanations about the study. Each patient was required to designate one primary caregiver, who was defined as a family member or friend providing the most unpaid care and support during the perioperative period and after discharge. The primary caregivers of postoperative thyroid cancer patients served as the study subjects.

Inclusion criteria were: age ≥ 18 years; undertaking the main caregiving responsibilities for the patient, with daily care duration ≥ 4 h; absence of serious physical illness; ability to read, write, and speak Chinese with normal communication skills. Exclusion criteria were: paid caregivers; individuals unable to communicate normally or with communication abnormalities; those unable to complete follow-up assessments.

All included primary caregivers were asked about their willingness to participate and provided written informed consent. The study was approved by the Ethics Committee of West China Hospital of Sichuan University [Approval No. 2020 (737)]. All research procedures were conducted in line with the Declaration of Helsinki.

### Sample size calculation

According to Kendall’s guidelines for sample size calculation, the survey sample size should be at least 5–10 times the number of independent variables (18). This study included a total of 55 explanatory variables, comprising 8 variables from the general demographic questionnaire, 20 variables from the Psychological Adaptation Scale (PAS), 22 variables from the Zarit Caregiver Burden Interview (ZBI), and 25 variables from the Family Caregiver Task Inventory (FCTI). Based on this principle, the estimated required sample size ranged from 275 to 550 participants. Taking into account a 20% non-response rate, a minimum of 344 participants were required. Based on the actual circumstances at our hospital, 387 cases were ultimately included in the analysis.

### Data collection

Data on primary caregivers, including age, gender, education level, employment status, marital status, relationship to the patient (spouse/non-spouse), cohabitation status, caregiving experience, availability of support, monthly household income per capita, and the patient’s medical expense payment method, were collected using a self-designed questionnaire. This questionnaire was administered once, 1 day before the patient’s discharge (T0).

Primary caregivers were assessed at four time points: T0 (1 day before discharge), T1 (1 month postoperatively), T2 (3 months postoperatively), and T3 (6 months postoperatively). Assessments were completed via electronic questionnaires or with telephone assistance. Of the 387 valid participants, 329 (85.0%) completed electronic questionnaires independently, while 58 (15.0%) completed the assessments via telephone assistance due to limited internet access or literacy concerns. To minimize potential mode-related bias, all research nurses received standardized training on uniform script reading and neutral prompting, and the same structured questionnaire with identical item order was used for both modes. No statistically significant differences were found between the two administration modes in PAS, ZBI, or FCTI scores at any time point (all *P* > 0.05), suggesting that data collection mode did not introduce substantial bias.

**FIGURE 1:**
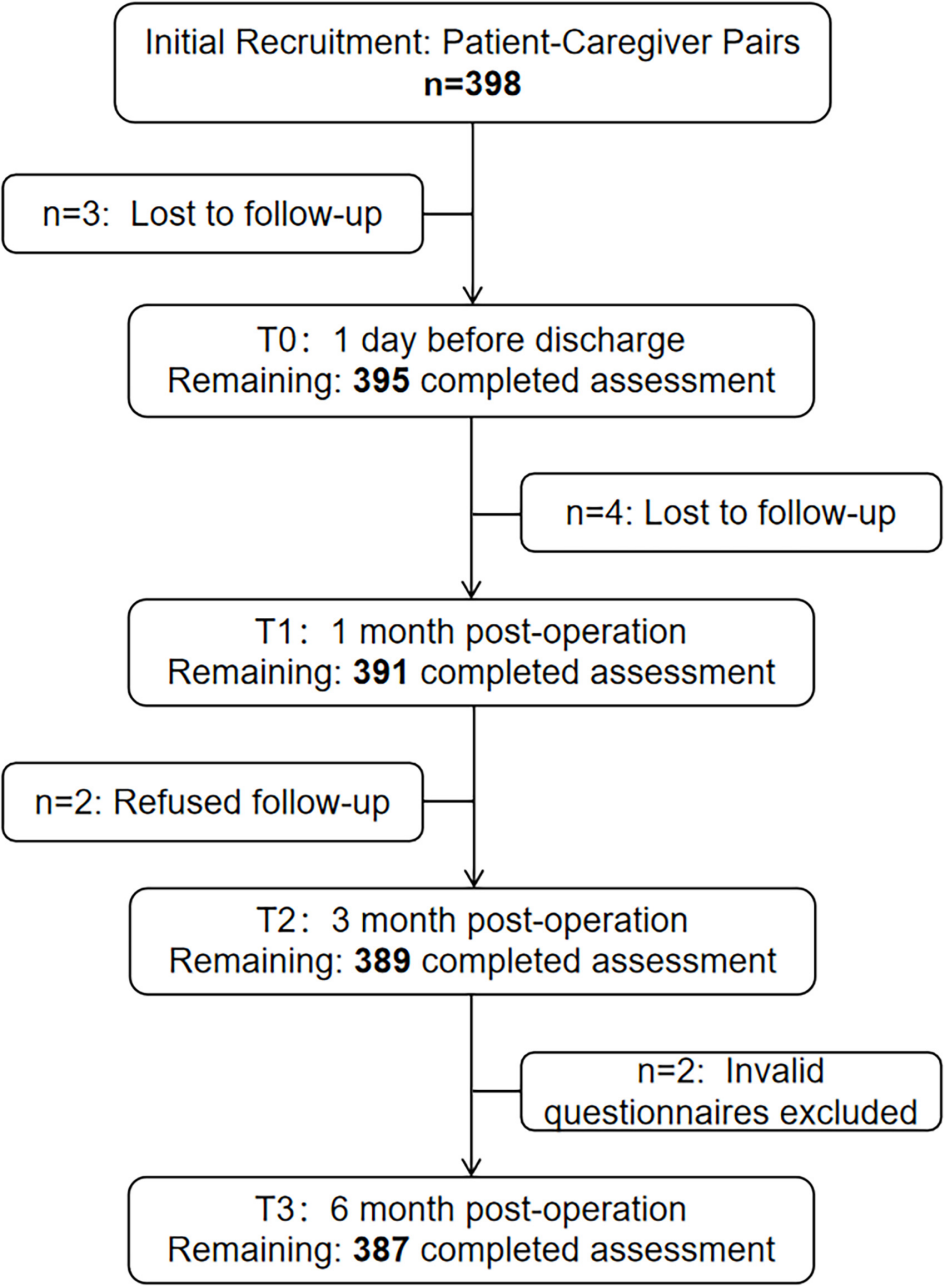
Research follow-up flowchart.

This study initially recruited 398 patient-caregiver pairs, with follow-up assessments conducted sequentially 1 day prior to discharge (T0), 1 month post-surgery (T1), 3 months post-surgery (T2) and 6 months post-surgery (T3); three participants were lost to follow-up at the T0 stage, with the remaining 395 completing the baseline assessment; At the T1 stage, 3 cases were lost to follow-up, with the remaining 392 completing the assessment; at the T2 stage, 2 invalid questionnaires (due to repetitive responses or contradictions) were excluded, leaving 390 cases; At the T3 stage, 3 cases were lost to follow-up due to invalid contact details; ultimately, 387 pairs were included in the statistical analysis. The total number of lost-to-follow-up and invalid cases was 11, resulting in an overall response rate of 97.23% (Figure 1).

### Questionnaire survey

PAS consists of 20 items across four dimensions: coping ability (4 items), self-strengthening (6 items), social ability (5 items), and psychological growth (5 items). The scale uses a 5-point Likert scale, with responses ranging from “Never” (scored 1) to “Almost Always” (scored 5). Total scores range from 20 to 100; higher scores indicate a better level of positive psychological adaptation in caregivers. Biesecker et al. developed the original scale in 2013 (19), and Mengjia et al. (20). translated and revised the Chinese version in 2021. The Chinese version has been extensively validated among primary carers of cancer patients in China (21). In the present study, the Cronbach’s α coefficient for the total PAS was 0.784. For the four subscales, Cronbach’s α values were as follows: coping ability (4 items) α = 0.76, self-strengthening (6 items) α = 0.81, social ability (5 items) α = 0.79, and psychological growth (5 items) α = 0.74, indicating acceptable to good internal consistency.

ZBI: Developed by Zarit et al. (22) in 1980, this scale has been widely used to measure caregiver burden (23), which has demonstrated good reliability and validity in assessing burden among postoperative caregivers of lung cancer patients in China (24). The ZBI consists of 22 items, each rated on a 5-point Likert scale from 0 (Never) to 4 (Nearly Always). The total score, obtained by summing all items, ranges from 0 to 88, with higher scores indicating greater burden. As per the classification by Hébert et al. (25), scores of 0–20 indicate little to no burden, 21–40 indicate mild to moderate burden, 41–60 indicate moderate to severe burden, and 61–88 indicate severe burden. In the present study, the ZBI showed a Cronbach’s α coefficient of 0.723.

FCTI: The FCTI comprises 25 items across five dimensions: adapting to the caregiving role, responding and providing assistance, managing personal emotional needs, assessing family and community resources, and adjusting personal life to caregiving demands, with each dimension containing 5 items. Responses are recorded on a 3-point Likert scale, where 0 represents not difficult, 1 represents difficult, and 2 represents very difficult. The total score ranges from 0 to 50, with a higher score indicating a lower level of caregiver competence. The scale was originally developed by Clark and Rakowski (26), and the Chinese version was translated and revised by Lee and Mok (15). In the present study, the Cronbach’s α coefficient for this scale was 0.753.

### Statistical methods

Data management and basic statistical analyses were performed using SPSS 25.0 and R version 4.5.2. Normally distributed continuous data are expressed as mean ± standard deviation (z̄ ± s). Missing data were handled by multiple imputation to minimize bias and improve estimation efficiency. Multicollinearity was examined using variance inflation factor (VIF) and tolerance before modeling. Normally distributed continuous data are expressed as mean ± standard deviation (z̄ ± s). Independent samples *t*-test or one-way ANOVA was used for comparisons between groups. Pearson correlation analysis was conducted to preliminarily examine the relationships among the variables. Repeated measures ANOVA was employed to compare scores across different time points. Hierarchical multiple linear regression and mediation effect analysis were performed to explore the influencing mechanisms and mediating pathways. Generalized Estimating Equations (GEE) were applied to identify longitudinal associated factors, accounting for within-subject correlations over time and providing regression coefficients (β) with 95% confidence intervals. Model diagnostics were conducted to ensure goodness-of-fit and robustness. A *P* < 0.05 was considered statistically significant.

## Results

### Comparison of PSA scores among primary caregivers with different sociodemographic characteristics

Analyses compared the psychological adaptation scores of primary caregivers across the four time points based on various sociodemographic characteristics (Table 1). The results revealed statistically significant differences in psychological adaptation scores over time based on caregiver age, education level, prior caregiving experience, number of caregivers, and monthly household income per capita (*P* < 0.05). Specifically, caregivers aged 41–59 and 60–68 generally reported higher adaptation scores than those aged 27–40. A higher education level was consistently associated with higher adaptation scores at all-time points. Caregivers with prior caregiving experience scored significantly higher than those without. Furthermore, caregivers with more helpers and those with a higher household income demonstrated significantly better psychological adaptation. In contrast, no statistically significant differences in psychological adaptation scores were found across the time points based on caregiver gender, marital status or relationship to the patient (*P* > 0.05).

**TABLE 1:** PSA scores of primary caregivers of postoperative thyroid cancer patients by general characteristics.

Characteristic	*n*	T0	T1	T2	T3
Gender					
Male	169	45.23 ± 5.04	48.95 ± 5.13	65.56 ± 6.68	70.29 ± 7.10
Female	218	45.12 ± 4.69	48.42 ± 5.14	65.87 ± 6.43	70.97 ± 7.33
*t*		0.215	0.945	−0.463	−0.880
*P*		0.830	0.345	0.644	0.379
Cohen’s d (95%CI)		0.021 (−0.18,4 0.225),	0.105 (−0.092, 0.303),	0.054 (−0.154, 0.252),	0.097 (−0.114, 0.295)
Age (years)					
27–40	138	43.95 ± 5.39	47.66 ± 5.21	63.91 ± 6.41	69.13 ± 7.73
41–59	192	45.88 ± 4.53	49.09 ± 5.10	66.51 ± 6.26	71.34 ± 6.85
60–68	57	45.73 ± 4.24	49.56 ± 4.89	67.54 ± 6.56	72.15 ± 6.84
*F*		6.65	3.90	8.61	4.79
*P*		0.001	0.021	< 0.001	0.009
η^2^ (95%CI)		0.034 (0.008, 0.068)	0.020 (0.003, 0.048)	0.044 (0.014, 0.082)	0.025 (0.005, 0.055)
Education level					
Middle high or below	244	43.88 ± 4.68	47.54 ± 4.68	63.94 ± 5.94	68.64 ± 6.66
High school	88	46.39 ± 4.01	49.31 ± 4.89	67.35 ± 5.71	72.66 ± 6.56
College or above	55	48.91 ± 4.60	52.52 ± 5.31	71.15 ± 6.35	76.50 ± 6.74
*F*		30.70	23.23	34.12	33.22
*P*		< 0.001	<0.001	< 0.001	<0.001
η^2^ (95%CI)		0.138 (0.090, 0.192)	0.109 (0.067, 0.159)	0.151 (0.101, 0.206)	0.148 (0.099, 0.203)
Marital status					
Married	360	45.22 ± 4.78	48.68 ± 5.19	65.62 ± 6.52	70.65 ± 7.33
Unmarried	27	44.52 ± 6.02	48.73 ± 4.76	67.33 ± 6.19	69.00 ± 6.01
*t*		0.93	−0.05	−1.34	1.25
*P*		0.353	0.960	0.181	0.212
Cohen’s d (95%CI)		0.134 (−0.273, 0.532)	0.011 (−0.398, 0.416)	0.267 (−0.144, 0.662)	0.236 (−0.174, 0.632)
Relationship to patient					
Spouse	174	44.98 ± 4.94	48.47 ± 5.04	65.77 ± 6.50	70.20 ± 7.04
Non-spouse	213	45.32 ± 4.84	48.72 ± 5.12	65.69 ± 6.28	70.70 ± 7.42
*t*		−0.67	−0.47	0.12	−0.68
*P*		0.502	0.638	0.904	0.497
Cohen’s d (95%CI)		0.074 (−0.137, 0.272)	0.054 (−0.153, 0.275)	0.01 3 (−0.192, 0.217)	0.074 (−0.132, 0.271)
Previous caregiving experience					
Yes	76	46.44 ± 4.95	50.08 ± 5.17	67.88 ± 7.13	73.38 ± 6.62
No	311	44.85 ± 4.81	48.30 ± 5.05	65.21 ± 6.25	70.01 ± 7.26
*t*		2.62	2.71	3.18	3.59
*P*		0.009	0.007	0.002	< 0.001
Cohen’s d (95%CI)		0.331 (0.087, 0.584),	0.351 (0.101, 0.640)	0.410 (0.155, 0.645)	0.484 (0.23,5 0.739)
Number of helpers					
0	242	44.34 ± 4.76	47.42 ± 4.65	64.83 ± 6.26	69.69 ± 7.08
1	92	46.00 ± 4.88	48.81 ± 4.17	66.21 ± 6.74	71.48 ± 7.88
≥ 2	53	47.44 ± 4.60	53.91 ± 5.25	69.02 ± 6.16	73.71 ± 5.91
*F*		10.75	40.15	9.08	7.05
*P*		< 0.001	<0.001	< 0.001	0.001
η^2^ (95%CI)		0.053 (0.023, 0.090)	0.173 (0.121, 0.229)	0.045 (0.018, 0.080)	0.035 (0.011, 0.067)
Monthly household income per capita (CNY)					
< 3,000	126	43.31 ± 5.05	45.96 ± 4.18	64.38 ± 6.72	69.00 ± 7.69
3,000–5,000	179	45.83 ± 4.73	48.04 ± 4.06	65.91 ± 6.86	70.89 ± 7.51
> 5,000	82	46.58 ± 4.10	54.10 ± 4.31	67.43 ± 4.85	72.75 ± 5.17
*F*		14.70	92.35	5.29	6.38
*P*		< 0.001	<0.001	0.005	0.002
η^2^ (95%CI)		0.071 (0.036, 0.113)	0.324 (0.257, 0.392	0.027 (0.006, 0.059)	0.032 (0.009, 0.065)

### Comparison of PAS, FCTI, and ZBI scores among primary caregivers across different time points

**FIGURE 2:**
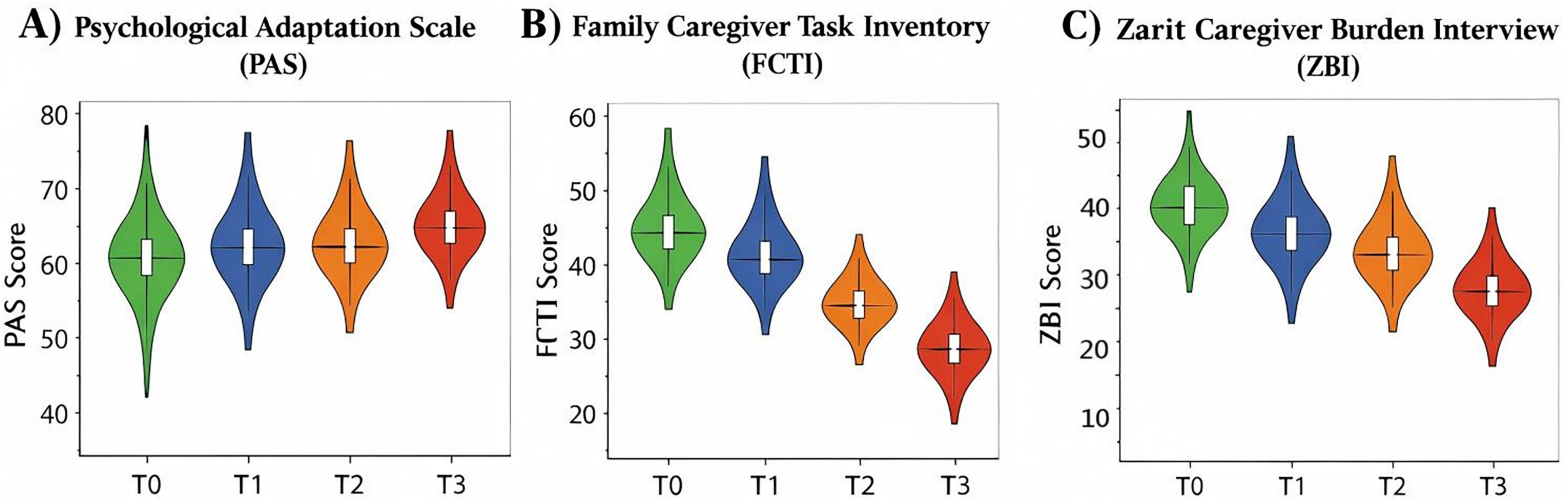
PAS, FCTI, and ZBI scores among primary caregivers across different time points. **(A)** PAS score. **(B)** FCTI score; **(C)** ZBI score.

The longitudinal changes in caregivers’ psychological adaptation, caregiving competence, and burden across the four assessment time points are summarized in Table 2. Repeated measures analysis of variance revealed significant changes over time in the scores of all three scales (*P* < 0.001). Scores on the PAS increased progressively from T0 to T3, indicating a continuous improvement in caregivers’ psychological adjustment over the 6-month postoperative period. In contrast, scores on both the FCTI and ZBI decreased significantly over time. The decline in FCTI scores reflects a reduction in caregivers’ perceived difficulty of caregiving tasks and an increase in caregiving competence, while the decrease in ZBI scores indicates a gradual alleviation of caregiver burden during the patient’s recovery phase (Figure 2).

**TABLE 2:** Comparison of PAS, FCTI, and ZBI scores among primary caregivers across different time points.

Scale	T0	T1	T2	T3	*F*	*P*	Partial η ^2^ (95% CI)
**PAS**	**45.28 ± 4.92**	**48.32 ± 5.19**	**66.41 ± 6.52**	**70.34 ± 7.38**	**1448.726**	**< 0.001**	0.791 (0.775, 0.817)
Coping ability	8.5 ± 1.2	9.3 ± 1.4	13.5 ± 1.8	14.2 ± 1.9			
Self-strengthening	13.2 ± 1.8	14.1 ± 2.0	19.8 ± 2.3	21.0 ± 2.5			
Social ability	11.1 ± 1.5	11.9 ± 1.6	16.8 ± 2.0	17.6 ± 2.2			
Psychological growth	12.48 ± 1.6	13.02 ± 1.7	16.31 ± 2.1	17.54 ± 2.3			
**FCTI**	**46.48 ± 8.68**	**41.18 ± 7.41**	**38.38 ± 7.27**	**34.43 ± 6.68**	**162.311**	**< 0.001**	0.304 (0.265, 0.343)
Adapting to caregiving role	10.2 ± 2.5	8.8 ± 2.1	8.0 ± 1.9	7.1 ± 1.7			
Providing assistance	9.8 ± 2.3	8.5 ± 2.0	7.9 ± 1.8	7.0 ± 1.6			
Managing emotional needs	9.5 ± 2.2	8.3 ± 1.9	7.7 ± 1.7	6.9 ± 1.5			
Assessing resources	8.9 ± 2.0	8.0 ± 1.8	7.6 ± 1.7	6.8 ± 1.4			
Adjusting personal life	8.08 ± 1.9	7.58 ± 1.7	7.18 ± 1.6	6.63 ± 1.4			
**ZBI**	**42.38 ± 7.79**	**39.20 ± 8.11**	**38.70 ± 6.49**	**33.92 5.56**	**7.892**	**< 0.001**	0.024 (0.011, 0.038)

The bolded rows represent the total scores of PAS, FCTI, and ZBI (mean ± SD), and the corresponding *F*, *P* indicate the differences in these total scores across the four time points (T0, T1, T2, and T3) by repeated-measures ANOVA.

### Correlation analysis between PAS and FCTI as well as ZBI scores

**FIGURE 3:**
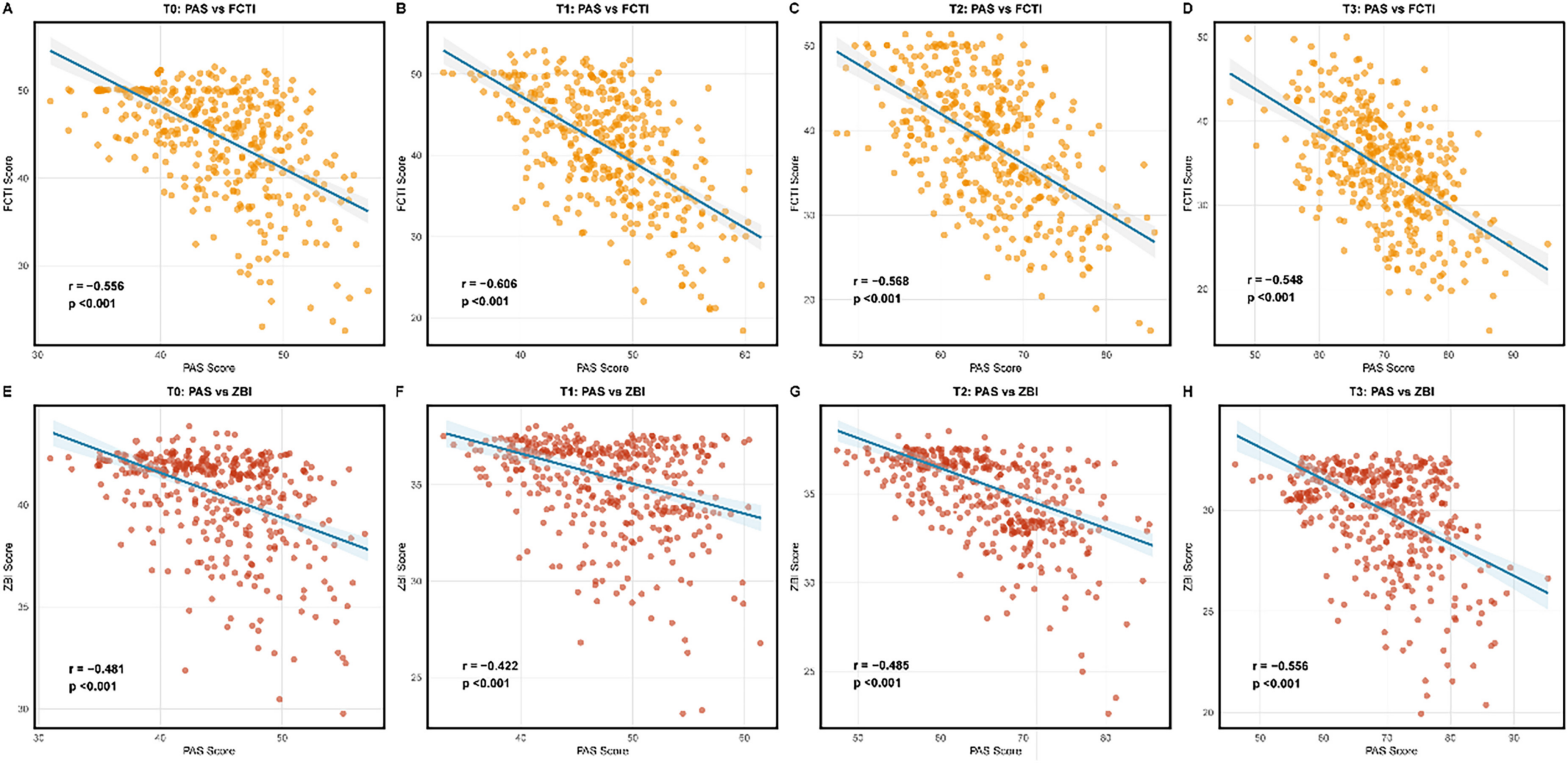
Correlation analysis. **(A)** T0: PAS vs. FCTI correlation. **(B)** T1: PAS vs. FCTI correlation. **(C)** T2: PAS vs. FCTI correlation. **(D)** T3: PAS vs. FCTI correlation; **(E)** T0: PAS vs. ZBI correlation. **(F)** T1: PAS vs. ZBI correlation. **(G)** T2: PAS vs. ZBI correlation. **(H)** T3: PAS vs. ZBI correlation.

To investigate the correlation between PAS, FCTI, and ZBI at different time points (T0, T1, T2, T3) for primary caregivers of postoperative thyroid cancer patients, Pearson correlation analysis was conducted. At each time point, a negative correlation between PAS and FCTI was observed (Figures 3A–D). As time progressed, the gap between caregivers’ psychological adaptation scores and their caregiver capacity scores increased. Similarly, negative correlations between PAS and ZBI were found, suggesting that as caregivers’ psychological adaptation scores improved, their caregiver burden gradually decreased (Figures 3E–H).

### Covariance test

A covariance analysis was conducted with PAS as the dependent variable and the following independent variables: variables showing statistically significant differences in PAS related general demographic information (age, education level, previous caregiving experience, number of caregivers, and monthly household income per capita), as well as ZBI and FCTI. As shown in Table 3, the VIF values for all independent variables were < 5 and the tolerance values were > 0.2 across all time points, indicating that there was no multicollinearity.

**TABLE 3:** Co-linearity test.

Variable	Tolerance	VIF
Age	0.719	1.341
Education level	0.778	1.256
Previous caregiving experience	0.814	1.268
Number of helpers	0.918	1.117
Monthly household income per capita	0.587	2.008
ZBI	0.574	2.108
FCTI	0.881	1.120

### Multiple linear hierarchical regression analysis of factors influencing PAS

Table 4 shows the assignment of values to the independent variables. The results of the hierarchical multiple regression analysis (Table 5) show that, in Model 1, which included socio-demographic variables, monthly per capita household income (> 5,000 yuan) was a significant positive predictor of PAS (β = 0.412, *P* < 0.001). Having one or more caregivers significantly improved adaptation levels compared with having no caregivers (β = 0.328, *P* = 0.021). Model 1 explains 18.6% of the variance in psychological adaptation. After introducing the ZBI into Model 2, the model’s explanatory power increased significantly (Δ*R*^2^ = 0.178, *P* < 0.001). The ZBI was a negative predictor of PAS-T3 (β = -0.286, *P* < 0.001), indicating that the heavier the caregiving burden, the poorer the psychological adaptation. The effect of having one caregiver remained significant (β = 0.291, *P* = 0.033), as did monthly per capita household income (> 5,000 yuan) (β = 0.227, *P* = 0.003). In Model 3, which further included FCTI, the model explained 51.2% of the variance in PAS (*R*^2^ = 0.512). FCTI was a negative predictor (β = -0.368, *P* < 0.001), as was ZBI (β = -0.271, *P* < 0.001).

**TABLE 4:** Specific assignments of independent variables and related dummy variables.

Variables	Specific assignment of independent variables and related dummy variables
Age	27–40 year (Z1 = 0, Z2 = 0, Z3 = 0, Z4 = 0) 41–59 year (Z1 = 0, Z2 = 1, Z3 = 0, Z4 = 0) 60–68 year (Z1 = 0, Z2 = 0, Z3 = 1, Z4 = 0)
Education level	Middle high or below (Z1 = 0, Z2 = 0, Z3 = 0, Z4 = 0) High school (Z1 = 0, Z2 = 1, Z3 = 0, Z4 = 0) College or above (Z1 = 0, Z2 = 0, Z3 = 1, Z4 = 0)
Previous caregiving experience	Yes = 1, No = 2
Number of caregivers	0 (Z1 = 0, Z2 = 0, Z3 = 0, Z4 = 0) 1(Z1 = 0, Z2 = 1, Z3 = 0, Z4 = 0) ≥ 2(Z1 = 0, Z2 = 0, Z3 = 1, Z4 = 0)
Monthly household income per capita	< 3,000(Z1 = 0, Z2 = 0, Z3 = 0, Z4 = 0) 3,000–5,000(Z1 = 0, Z2 = 1, Z3 = 0, Z4 = 0) > 5,000(Z1 = 0, Z2 = 0, Z3 = 1, Z4 = 0)
ZBI	Raw value input
FCTI	Raw value input

**TABLE 5:** Multiple linear hierarchical regression analysis of factors influencing PAS (T3).

Variables	Intra-class predictor variables	Model 1			Model 2			Model 3		
		β	95%CI	*P*	β	95%CI	*P*	B	95%CI	*P*
Age	41–59 year	0.108	(−0.124, 0.340)	0.355	0.083	(−0.149, 0.315)	0.472	0.071	(−0.159, 0.301)	0.543
	60–68 Year	0.132	(−0.218, 0.482)	0.459	0.105	(−0.246, 0.456)	0.561	0.092	(−0.256, 0.440)	0.605
Education level	High school	0.116	(−0.182, 0.414)	0.443	0.094	(−0.207, 0.395)	0.538	0.081	(−0.215, 0.377)	0.582
	College or above	0.141	(−0.179, 0.461)	0.387	0.123	(−0.202, 0.448)	0.453	0.109	(−0.214, 0.432)	0.507
Number of helpers	1	0.328	(0.051, 0.605)	0.021	0.291	(0.024, 0.558)	0.033	0.274	(0.012, 0.536)	0.041
	≥ 2	0.085	(−0.236, 0.406)	0.594	0.062	(−0.255, 0.379)	0.699	0.051	(−0.270, 0.372)	0.758
Monthly household income per capita	3,000–5,000	0.297	(0.064, 0.530)	0.013	0.184	(−0.043, 0.411)	0.111	0.169	(−0.056, 0.394)	0.143
	> 5,000	0.412	(0.178, 0.646)	< 0.001	0.227	(−0.003, 0.457)	0.053	0.211	(−0.017, 0.439)	0.074
ZBI		−	−	−	−0.286	(−0.354, −0.218)	< 0.001	−0.271	(−0.339, −0.203)	< 0.001
FCTI		–	–	–	–	–	–	−0.368	(−0.427, −0.309)	< 0.001
Regression model	*F*	5.821		0.001	12.374		< 0.001	16.529		< 0.001
	*R* ^2^	0.186			0.364			0.512		
	Adjusted *R*^2^	0.162			0.347			0.498		
	Δ*R*^2^	—			0.178			0.148		

### Factors associated with PAS in primary caregivers

**FIGURE 4:**
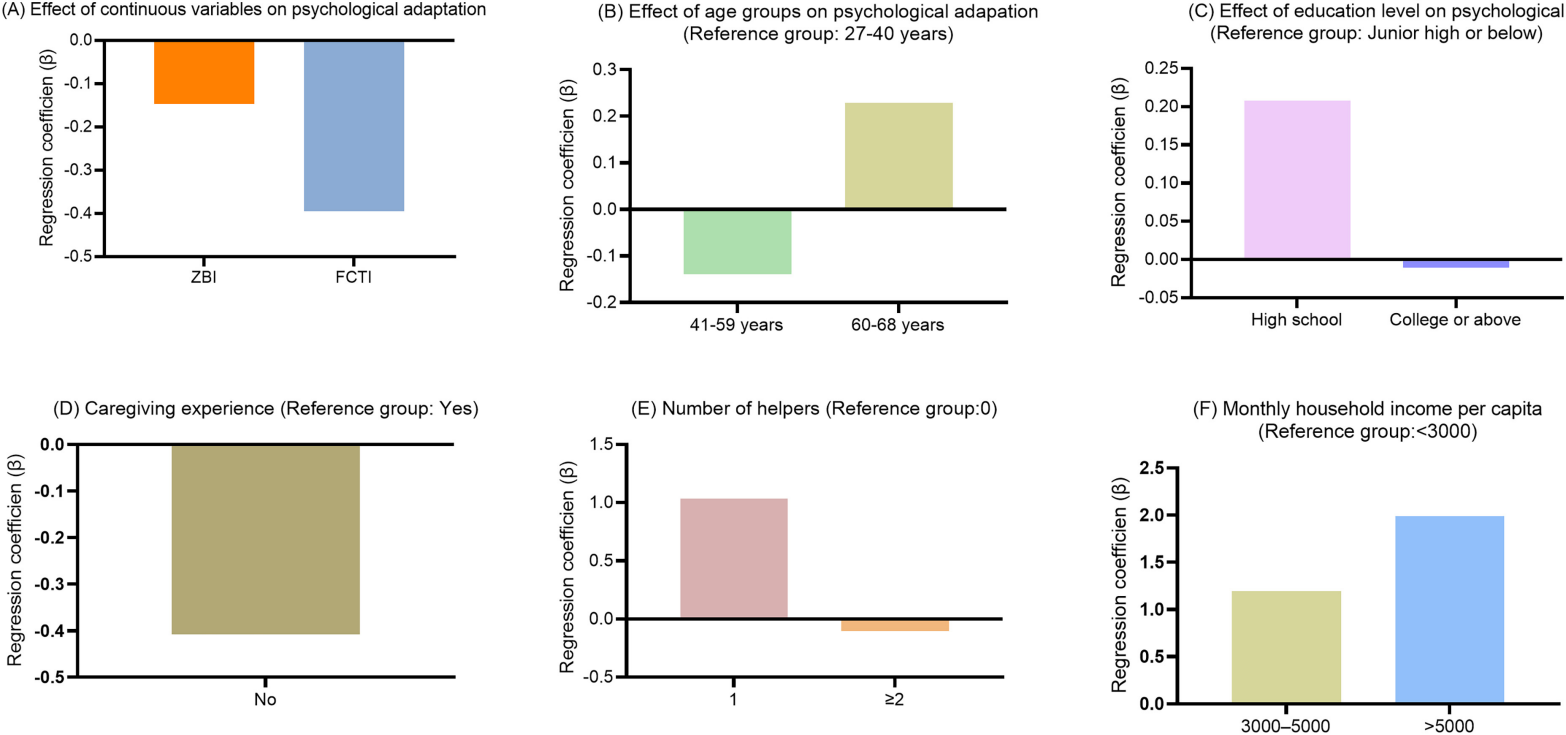
The trends of pas in relation to key variables. **(A)** Effect of continuous variables on pas. **(B)** Effect of age groups on psychological adapation (Reference group: 27-40 years). **(C)** Effect of education level on psychological (Reference group: Junior high or below). **(D)** Caregiving experience (Reference group: Yes). **(E)** Number of helpers (Reference group: 0). **(F)** Monthly household income per capita (Reference group: < 3,000).

This study employed GEE to analyze factors associated with psychological adaptation in caregivers of postoperative thyroid cancer patients. As shown in Table 6, time (from T0 to T3) had a significant positive effect on psychological adaptation (β = 7.928, *P* < 0.001), indicating that caregivers’ psychological adaptation improved progressively over the postoperative period. Having one caregiver was found to significantly improve psychological adaptation compared to having none (β = 1.035, *P* = 0.014), suggesting that moderate support is beneficial. Compared with a monthly household income per capita of < 3,000, there was a marked improvement in psychological adjustment levels for those with incomes of 3,000–5,000 and over 5,000 (β = 1.192, *P* = 0.009; β = 1.987, *P* < 0.001), suggesting that higher income has a significant positive impact. ZBI was negatively associated with psychological adaptation (β = -0.147, *P* < 0.001), suggesting that higher burden corresponded to lower adaptation levels. FCTI showed a negative association with adaptation (β = -0.395, *P* < 0.001), indicating that higher FCTI scores were linked to worse psychological adaptation. Figure 4 further illustrates the trends of psychological adaptation in relation to key variables.

**TABLE 6:** Factors associated with pas in primary caregivers.

Variable	β (95% CI)	SE	*t*	*P*
Intercept	53.182 (50.118, 56.246)	1.565	34.000	< 0.001
Time (per unit increase from T0 to T3)	7.928 (7.631, 8.225)	0.151	52.503	< 0.001
Age group (ref: 27-40 years)				
41–59 years	−0.139 (−0.921, 0.643)	0.399	−0.348	0.728
60–68 years	0.229 (−0.991, 1.449)	0.622	0.368	0.713
Education level (ref: Junior high or below)				
High school	0.208 (−0.654, 1.070)	0.440	0.473	0.636
College or above	−0.011 (−1.002, 0.980)	0.506	−0.022	0.983
Caregiving experience (ref: Yes)				
No	−0.408 (−1.310, 0.494)	0.460	−0.887	0.375
Number of helpers (ref: 0)				
1	1.035 (0.208, 1.862)	0.422	2.453	0.014
≥ 2	−0.102 (−1.216, 1.012)	0.568	−0.180	0.857
Monthly household income per capita (Ref: < 3,000)				
3,000–5,000	1.192 (0.298, 2.086)	0.456	2.614	0.009
> 5,000	1.987 (1.040, 2.934)	0.483	4.114	< 0.001
ZBI	−0.147 (−0.180, −0.114)	0.017	−8.647	< 0.001
FCTI	−0.395 (−0.445, −0.345)	0.025	15.800	< 0.001

### Sensitivity analyses

To examine the impact of time-related factors on the results, this study conducted a GEE sensitivity analysis by replacing the continuous time variable with a categorical time variable (using T0 as the reference point). The results showed (Table 7) that, compared with the period prior to discharge (T0), the caregivers’ psychological adaptation levels exhibited a significant upward trend at 1 month (T1), 3 months (T2), and 6 months (T3) post-surgery (all *P* < 0.05). At the same time, there were no significant changes in the direction of effect, effect size or statistical significance of the number of people cared for, monthly per capita income, ZBI and FCTI on psychological adaptation; the association patterns between these variables and psychological adaptation remained stable.

**TABLE 7:** Sensitivity analysis.

Variable	β (95% CI)	SE	*P*
Intercept	52.874 (50.012, 55.736)	1.582	< 0.001
Time (ref: T0)			
T1	3.042 (2.516, 3.568)	0.261	< 0.001
T2	21.135 (20.427, 21.843)	0.352	<0.001
T3	25.061 (24.354, 25.768)	0.353	< 0.001
Age group (ref: 27–40 years)			
41–59 years	−0.126 (−0.898, 0.646)	0.393	0.745
60–68 years	0.217 (−0.985, 1.419)	0.618	0.721
Education level (ref: Junior high or below)			
High school	0.195 (−0.647, 1.037)	0.436	0.648
College or above	−0.008 (−0.986, 0.970)	0.502	0.987
Caregiving experience (ref: Yes)			
No	−0.401 (−1.287, 0.497)	0.451	0.382
Number of helpers (ref: 0)			
1	1.026 (0.201, 1.851)	0.422	0.014
≥ 2	−0.094 (−1.208, 1.020)	0.564	0.861
Monthly household income per capita (Ref: < 3,000)			
3,000–5,000	1.183 (0.291, 2.075)	0.452	0.010
> 5,000	1.974 (1.031, 2.917)	0.479	< 0.001
ZBI	−0.145 (−0.178, −0.112)	0.014	< 0.001
FCTI	−0.392 (−0.442, −0.342)	0.021	< 0.001

## Discussion

Thyroid cancer has become one of the most prevalent malignant tumors worldwide, with a persistently rising incidence (27). Although its prognosis is relatively favorable, postoperative rehabilitation involving long-term medication, functional symptom management, and recurrence prevention imposes sustained care demands on family caregivers (3). This 6-month longitudinal study focused on primary caregivers of postoperative thyroid cancer patients, exploring the dynamic trajectory of psychological adaptation and its associations with caregiver burden and caregiving competence.

In the present study, the psychological adaptation scores of primary caregivers increased continuously from discharge preparation (T0) to 6 months postoperatively (T3), accompanied by declining scores on the ZBI and FCTI. This trend suggests that caregivers undergo a gradual adaptive process alongside patients’ postoperative recovery. At the perioperative stage, caregivers face unfamiliar care tasks, uncertainty about the disease, and emotional stress, resulting in lower adaptation levels and higher perceived burden and difficulty. As time passes, caregivers become familiar with care procedures, master symptom management skills, and witness patients’ stable recovery, thereby reducing anxiety and enhancing self-efficacy. This dynamic pattern is consistent with previous longitudinal findings in cancer caregivers (4), confirming that psychological adaptation is a time-dependent progressive recovery process rather than a static state. It is worth noting that this study found the most significant improvement in psychological adjustment scores occurred between 1 and 3 months post-surgery, suggesting that this period represents a critical window for psychological adjustment. Consequently, clinical support should be intensified during this transitional phase to consolidate the caregivers’ adaptive progress.

Correlation analysis revealed a negative correlation between psychological adaptation and caregiver burden and caregiving difficulty at all four time points, with these associations strengthening over time. Higher caregiver burden and lower perceived competence corresponded to poorer psychological adaptation, which is consistent with cross-sectional evidence in breast and lung cancer caregivers (24, 28). Although the prognosis for thyroid cancer patients is better than for many other malignancies, the long-term, detailed nature of postoperative care, including medication adherence, voice and neck symptom monitoring, and dietary management, still generates cumulative stress (29, 30). When caregivers perceive a heavy burden or insufficient competence, they are more likely to experience emotional exhaustion, reduced coping ability and impaired social and personal growth, all of which hinder psychological adaptation. Conversely, lower burden and higher competence enable caregivers to maintain stable emotions and positive thinking, thereby promoting better adaptation. These results emphasize that burden and competence are key psychological factors closely linked to adaptation throughout the caregiving process.

GEE analysis further identified time, number of co-caregivers, caregiver burden, and caregiving competence as significant predictors of psychological adaptation. As the follow-up period progressed from T0 to T3, the psychological adaptation level of caregivers showed a significant upward trend, suggesting that as the postoperative rehabilitation process extended, the psychological adaptation of caregivers might show a gradually improving trend. This trend may be related to caregivers gradually becoming familiar with the care process, improving their care skills, and the patient’s condition stabilizing, and is consistent with previous research results showing that the psychological adaptation of cancer caregivers gradually improves over time (31). In terms of the number of helpers, caregivers with one co-caregiver had a significantly higher level of psychological adaptation than those without assistance. Having two or more caregivers did not provide any additional significant benefits. One possible explanation is that multiple caregivers may lead to unclear division of caregiving responsibilities and increased coordination costs, which could offset the potential benefits of additional support. However, the lack of statistical significance for the ≥ 2 caregiver group may also be attributable to the relatively small sample size in this subgroup (*n* = 53) and the single-center setting. Future multi-center studies with larger samples are needed to explore the shape of this relationship. In terms of family economic factors, compared with caregivers whose family’s average monthly income was < 3,000 yuan, those with an income of 3,000–5,000 yuan and more than 5,000 yuan had a higher psychological adaptation level, suggesting that relatively better economic conditions may provide more sufficient resource guarantees for caregivers, thereby facilitating psychological adaptation. This result may reflect that economic pressure is a potential factor affecting the psychological state of the care group, and is consistent with previous research results on cancer family caregivers (32). In terms of negative factors, the higher the score of the ZBI, the lower the psychological adaptation level, indicating that excessive care burden may consume the psychological resources of caregivers and have an adverse impact on psychological adaptation. At the same time, the score of the FCTI was higher, representing lower care ability, and it was significantly negatively correlated with psychological adaptation, suggesting that the caregivers’ self-perceived lack of care ability may be an important factor restricting their psychological adaptation. The above results to some extent support that care burden and care ability are key variables affecting psychological adaptation in the cancer care scenario. In this study, factors such as age, educational attainment and previous caregiving experience did not show statistically significant differences. This may be attributed to the single-center nature of the sample, the relative concentration of the geographical area and healthcare setting, and the relatively low intensity of care required for thyroid cancer. Furthermore, the influence of age, educational attainment and caregiving experience on psychological adjustment may not be independent; rather, it may be the result of the combined influence of various factors, including the passage of time, social support and economic circumstances. Further exploration can be conducted through multi-center, large-sample designs in the future.

To deeply investigate the hierarchical influence of various factors on psychological adaptation 6 months after surgery (at time point T3), this study conducted a multivariate hierarchical regression. Model 1 only included socio-demographic variables. The results showed that a monthly per capita household income of more than 5,000 yuan and having one helpers had a significant positive predictive effect on psychological adaptation. This finding is consistent with the results of studies on cancer caregivers, indicating that economic resources are an important external factor influencing the psychological condition of caregivers (33). After introducing the ZBI into Model 2, the explanatory power of the model significantly increased, and ZBI showed a significant negative predictive effect, suggesting that the increase in care burden may directly consume psychological resources and reduce the level of adaptation, becoming a key negative factor that restricts psychological adaptation. After further incorporating the FCTI into Model 3, the explanatory power of the model increased again. FCTI also showed a significant negative predictive effect, and its strength was higher than that of ZBI. This indicates that the perceived care capacity by the caregivers themselves may be a more core influencing factor than the burden, and it also suggests that care capacity may play a dominant role in psychological adaptation.

Model 1, Model 2, and Model 3, respectively explain 18.6, 36.4, and 51.2% of the variance in psychological adaptation. After including the burden of care, the explanatory power increased from 18.6 to 36.4%; further adding the care capacity, the explanatory power rose to 51.2%. Although the model fit has improved, due to the limitations of the variables included in this study, its overall explanatory power still has certain limitations. The absence of measured related variables may affect the explanatory degree of the model. For example, previous studies have shown that factors such as social support, family resilience, coping styles, and social resources can further enhance the explanatory power of the psychological adaptation model for caregivers (34).

## Limitations

Although this study provides empirical evidence regarding the psychological adjustment of primary carers following thyroid cancer surgery, it has certain limitations. Firstly, the study employed a single-center longitudinal design, with the sample drawn exclusively from West China Hospital. As a large Grade A tertiary hospital in western China, this institution differs significantly from other regions or primary-care and rural medical institutions in terms of clinical standards, rehabilitation guidance, social support and economic conditions; consequently, the findings cannot be directly generalized to carer groups in different healthcare settings across the country. Future research should adopt a multi-center, large-sample design, incorporating caregivers from different provinces, institutional levels and rural areas. Secondly, the data are based on self-reports by caregivers, which are susceptible to social expectations and reporting biases. Furthermore, the study focused solely on informal primary caregivers and did not include paid or professional nursing staff; although a protective effect was observed for co-caregivers, the quality, frequency and task distribution of their support were not measured. Future research could compare purely informal care settings with those involving paid assistance, and assess the contributions of co-carers in greater detail. Finally, the analysis relied on caregivers’ subjective perceptions and did not incorporate objective indicators such as the patient’s tumor stage, postoperative complications (e.g., hypoparathyroidism, recurrent laryngeal nerve injury) or radiotherapy requirements. Subsequent studies should combine subjective and objective variables to elucidate the dynamic impact of the patient’s clinical progression on the psychological adaptation of caregivers.

## Conclusion

This 6-month longitudinal study of primary caregivers of patients after thyroid cancer surgery yielded the following findings. From discharge preparation (T0) to 6 months postoperatively (T3), caregiversary caregivers of patients after thyroid cancer surgery yielded the following findings. objective variabl Correlation analyses at all four time points showed negative associations between psychological adaptation and both caregiver burden and caregiving difficulty, with these associations strengthening over time. GEE analysis identified time, number of co-caregivers, caregiver burden, and caregiving competence as significant predictors of psychological adaptation. Specifically, caregivers with one helper had higher psychological adaptation levels than those without any assistance; having two or more co-caregivers did not confer additional significant benefits. Higher monthly household income (3,000–5,000 yuan and > 5,000 yuan vs. < 3,000 yuan) was associated with higher psychological adaptation scores. Higher ZBI scores and higher FCTI scores were associated with lower psychological adaptation. In hierarchical regression analyses, the final model (Model 3), which included socio-demographic variables, ZBI, and FCTI, explained 51.2% of the variance in psychological adaptation at 6 months postoperatively. The FCTI showed a stronger negative predictive effect than the ZBI in this model.

## Data Availability

The raw data supporting the conclusions of this article will be made available by the authors, without undue reservation.
